# 

**Published:** 1858-05

**Authors:** 


					﻿PUBLISHED MONTHLY.
! VOL. L]
[No. 5.
C IC4Oo
MEDICAL JOURNAL
' EDITfeD BY
TST_ S_ DAVIS, 3\ZE_ ZD_3
AND
TSE- B'VFORD, 2ME_1D.
WAY, 1858.
JAS. BARNET, BOOK AND JOB PRINTER, “CHRONOTYPE” OFFICE,
189 Lake Street, (Corner of Wells), Chicago, Ill.
AT $2 PEB ANNUM, IN ADVANCE.
				

